# Iodine nutritional status and its associations with thyroid function of pregnant women and neonatal TSH

**DOI:** 10.3389/fendo.2024.1394306

**Published:** 2024-05-31

**Authors:** Lijun Fan, Ye Bu, Shiqi Chen, Sihan Wang, Wei Zhang, Yan He, Dianjun Sun

**Affiliations:** ^1^ Endemic Disease Control Center, Chinese Center for Disease Control and Prevention, Harbin Medical University, Harbin, China; ^2^ National Health Commission, Education Bureau of Heilongjiang Province, Key Laboratory of Etiology and Epidemiology (23618504), Harbin, China; ^3^ Heilongjiang Provincial Key Laboratory of Trace Elements and Human Health, Harbin Medical University, Harbin, China; ^4^ The Fourth Affiliation Hospital, Harbin Medical University, Harbin, China

**Keywords:** pregnant women, iodine nutrition, thyroid disorders, neonatal outcome, TSH

## Abstract

**Introduction:**

Iodine serves as a crucial precursor for the synthesis of thyroid hormones and plays an import role in both pregnant women and their offspring. The relationships between iodine nutritional status and maternal thyroid function and neonatal outcomes remain inconclusive in areas with adequate iodine nutrition. This study aims to investigate their correlations.

**Methods:**

Blood, morning urine and 24-hour urine were collected from the pregnant women to measure thyroid functions, serum iodine concentration (SIC), morning urine iodine concentration (UIC) and 24-hour urine iodine excretion (24-hour UIE). Indicators of their offspring’s neonatal indexes were recorded.

**Results:**

A total of 559 pregnant women were enrolled in this study. The iodine indicators including Tg, 24-hour UIE and morning UIC were significantly different among the euthyroid pregnant women and those with different thyroid disorders. The levels of FT3, FT4, and SIC exhibited a gradual decline and the concentration of TSH exhibited a gradual increase trend throughout the progression of pregnancy in euthyroid pregnant women. There were no significant differences in neonatal outcomes and neonatal TSH values among euthyroid pregnant women and thyroid disorders pregnant women. SIC had a significant impact on maternal FT4 levels throughout all three trimesters, with varying degrees of importance observed in each trimester. TSH level emerged as the primary determinant of FT4 during the first trimester, while SIC exerted a predominant influence on FT4 levels in the second and third trimesters. The prevalence of thyroid disorders in pregnant women was the lowest when the SIC of pregnant women was probable in the range of 60~70 μg/L, 24-hours UIE was in the range of 250~450 μg, and Tg was in the range of 9~21 μg/L. Maternal TSH exhibited a notable influence on neonatal TSH levels, particularly at the *50th* and *75th* quantiles. Among the iodine nutritional indicators, SIC and morning UIC demonstrated higher AUC values for abnormal FT4 and TSH, respectively.

**Discussion:**

The iodine nutrition status of pregnant women exerts an impact on their thyroid function and prevalence of thyroid disorders, and neonatal TSH was affected by maternal TSH. SIC may be a better indicator for iodine nutritional assessment than other indexes.

## 1 Introduction

Iodine is an essential micronutrient in human due to its role as a constituent of thyroid hormone. During pregnancy, there is a significant increase in iodine requirements primarily attributed to the augmented synthesis of maternal thyroid hormone, the rise in in renal iodine clearance, and the necessity to fulfill fetal growth and development demands (1, 2). In early pregnancy, maternal thyroid hormone plays a pivotal role to the fetal development, while in the middle and late stages, the maturation of fetal thyroid development facilitates the direct utilization of iodine for the synthesis of thyroid hormone, thereby facilitating growth and development.

Maintaining adequate iodine nutrition during pregnancy is of utmost importance due to the potential adverse effects that iodine deficiency and excess can have on pregnant women (3, 4). However, nowadays many studies only focused on the iodine nutritional status of pregnant women, and seldom studies concerned on the effect of iodine nutrition on the thyroid function of pregnant women and their neonatal outcome. Among these studies, one conducted in Shanghai (China) displayed that both low and high urine iodine concentration was related to thyroid function and autoimmunity (5). A cross-sectional study in the southeast coast of China showed that serum iodine concentration (SIC) negatively correlated with thyroid stimulating hormone (TSH), and positively correlated with other thyroid function indicators, which indicating SIC may serve as a composite indicator of iodine nutritional status (6). A study conduct in northern Spain indicated that the consumption of iodized salt was associated with adequate iodine nutrition but not with maternal thyroid function (7). Despite the above studies, studies on the association of iodine nutrition with thyroid function, especially with thyroid function related disease in pregnant women are still very limited.

Similarly, maintaining adequate iodine nutrition and thyroid function during pregnancy is also critical for perinatal outcomes. A study carried out in North Macedonia showed total serum thyroxine (TT4) had a statistically significant inverse predictive impact on low birth weight, and thyroglobulin (Tg) had a positive impact on low birth weight (8). A study in Australia showed no correlation between iodine intake and their neonates’ TSH (9). A study in iodine excess areas showed that excessive iodine intake during pregnancy was associated with an increased rate of hyperthyrotropinaemia in neonates and their mothers, especially in male neonates (10). However, the effects of moderate variations in maternal iodine nutrition on fetal thyroid function are poorly understood.

Several studies have proposed to assess iodine nutritional status in pregnant women using SIC, Tg, 24-hour urinary iodine excretion (24-hour UIE) or thyroid function (11–14). However, due to their variation and change, there remains an absence of uniform standards for normal reference ranges of these indicators in pregnant women in China, and even the worldwide. In addition, currently, over 190 countries worldwide have implemented universal salt iodization (USI) policy. China stands out as one of the nations with exemplary USI policy, and the consumption rate of qualified iodized salt can reach more than 90% nationwide. Presently, China has maintained an appropriate iodine nutritional status among the general population. Under this background, there remains a lack of clarity regarding the impact of thyroid function and iodine nutrition indicators on pregnant women and their association with neonatal TSH levels. The objective of this study is to investigate the iodine status and its associations with thyroid function in pregnant women and their offspring. This study will provide more evidence for the evaluation of iodine nutrition status in individual in pregnant women, and provide further scientific reference for the maintaining suitable iodine nutritional status in pregnant women in China.

## 2 Materials and methods

### 2.1 Study population

This prospective study recruited a total of 559 pregnant women who attended antenatal checkup at the Forth Affiliated Hospital of Harbin Medical University, Harbin, China, between January 2021 and December 2022. The results of China national iodine deficiency disorders surveillance in Harbin in recent years showed that the coverage rate of iodized salt was > 95%, the consumption rate of qualified iodized salt was > 90%, the median UIC of children aged 8 ~ 10 years was between 100 ~ 300 μg/L, and the median UIC of pregnant women was between 150 ~ 249 μg/L.

A comprehensive questionnaire was used to collect baseline information, including participants’ names, ages, gestational weeks, gravidity and parity status, as well as their medical history. The inclusion criteria encompassed women aged between 18 and 40 years old with a minimum residency period of five years in Harbin and a singleton pregnancy. Pregnant women with pre-existing documented thyroid or other chronic diseases, multiple pregnancies, or recent consumption of iodine-rich foods within three days were excluded from the study to prevent interference with iodine nutritional levels.

The study was approved by the ethics committees of Harbin Medical University (HRBMUCEDC20211002). This study was conducted according to the provisions of the Declaration of Helsinki. Written informed consent was obtained from all participants or their guardians.

### 2.2 Laboratory analysis

#### 2.2.1 Sample collection and processing

Fasting venous blood was also collected from all pregnant women in the morning. After separating the serum, 300 μL aliquots of each sample were stored at -80°C until further use. The morning urine and 24-hour urine was both collected. Researchers provided each participant with detailed instructions on the proper method for collecting 24-hour urine samples. Participants were instructed to empty their bladder at 6:00 am on the first day (as the morning urine), followed by collection of all subsequent urine until 6:00 am the next day. Complete urine samples were collected in clean polyethylene barrels provided by unification, and participants used cylinders to measure their 24-hour urine volume. After thorough mixing, a 5-ml sample of the 24-hour urine was placed into cryogenic tubes.

#### 2.2.2 Testing and analysis

SIC, morning urine iodine concentration (UIC), 24-hour UIE was determined by arsenic and cerium catalytic spectrophotometry recommended by WHO (15). TSH, free triiodothyronine (FT3), free thyroxine (FT4), thyroglobulin antibody (TgAb), thyroid peroxidase antibody (TPOAb) were tested using a chemiluminescent immunoassay (IT3000, Roche, Switzerland). Tg were determined by electrochemiluminescence (cobas 8000, Roche, Switzerland).

#### 2.2.3 Diagnostic criteria

The reference ranges of TSH were 0.09 ~ 4.52 mU/L in the first trimester, 0.45 ~ 4.32 mU/L in the second trimester, and 0.30 ~ 4.98 mU/L in the third trimester. The reference ranges of FT4 were 13.15 ~ 20.78 pmol/L in the first trimester, 9.77 ~ 18.89 pmol/L in the second trimester, and 9.04 ~ 15.22 pmol/L in the third trimester. The reference ranges of FT3, TgAb and TPOAb were 3.1 ~ 6.8 pmol/L, 0 ~ 115 U/mL, 0 ~ 34 U/mL (16).

The thyroid disorders in this study included hypothyroidism, subclinical hypothyroidism, hyperthyroidism, subclinical hyperthyroidism, hypothyroxinemia, hyperthyroxinemia, autoimmune thyroiditis in this study. The diagnostic criteria for thyroid disorders were as follows. Hypothyroidism: TSH above the upper limit and FT4 below the lower limit. Subclinical hypothyroidism: TSH above the upper limit and FT4 within the normal range. Hyperthyroidism: TSH below the upper limit and FT4 above the upper limit. Subclinical hyperthyroidism: TSH below the upper limit and FT4 within the normal range. Hypothyroxinemia: TSH within the normal range and FT4 below the lower range. Hyperthyroxinemia: TSH within the normal range and FT4 above the higher range. Autoimmune thyroid disease: TPOAb > 34 U/mL and/or TgAb > 115 U/mL (17).

### 2.3 Evaluation of the neonatal outcome

The neonatal outcomes of all pregnant women were monitored, encompassing the timing and mode of delivery. Additionally, comprehensive data on their offspring was collected, including gender, body length, weight, head circumference, Apaka score assessment results, as well as neonatal TSH screening outcomes.

### 2.4 Statistical analysis

In this study, categorical variables were represented as relative frequencies, continuous variables following a normal distribution were presented as means ± standard deviations, and continuous variables not conforming to a normal distribution were expressed as medians with *P25* and *P75*. The differences between groups for categorical variables were assessed using the chi-square test, while the t-test was employed for comparing normally distributed continuous variables. Nonparametric tests were utilized to compare non-normally distributed continuous variables. Spearman rank correlation was used for correlation analysis. Linear regression and quantile regression were used to analyze the influencing factors of continuous variables and for the test of trend. Logistics regression was used to analyze the influencing factors of categorical variables. The receiver operating characteristic (ROC) curve, and the area of receiver operating characteristic curve (AUC) values were performed to describe the discriminatory efficacy of different indicators. Statistical analysis was performed by SAS 9.1.3 and R platform. *P* ≤ 0.05 represented as a statistical significance.

## 3 Results

### 3.1 The thyroid function status and iodine nutrition level of the included pregnant women and the offspring

A total of 559 pregnant women were enrolled in this study, with 198, 217 and 144 participants in the first, second, and third trimester, respectively. Out of the total sample size, 429 pregnant women were found to be euthyroid while the remaining 130 participants exhibited thyroid disorders. The results are displayed in **Tables 1**, **2** (in **Table 2**, due to the low number of women suffering from different thyroid disorders, the comparisons of different indicators were not separated according to trimesters).

**Table 1:** Characteristics of the healthy pregnant women.

Indicator	The first trimester	The second trimester	The third trimester	*S*	*P ^trend^*
*n*	145	173	111	–	–
Age (year)	30.3 ± 3.4	31.0 ± 3.6	30.8 ± 4.3	–	–
Weight (kg)	61.2 ± 12.3	60.0 ± 10.6	59.4 ± 11.2	–	–
BMI (kg/m^2^)	22.9 ± 4.6	22.1 ± 4.8	22.3 ± 4.2	–	–
Pregnant week (week)	12.5 (12.0~13.0)	24.0 (17.5~26.0)	29.2 (27.4~33.1)	–	–
TSH (mU/L)	1.5 ± 1.0	2.0 ± 0.9	2.1 ± 1.0	16.0	<0.01
FT3 (pmol/L)	5.0 ± 0.5	4.5 ± 0.6	4.3 ± 0.5	75.1	<0.01
FT4 (pmol/L)	16.7 ± 1.7	13.5 ± 1.8	12.6 ± 1.5	215.4	<0.01
TgAb (U/mL)	15.3 (13.6~17.8)	15.1 (13.1~17.3)	14.9 (13.3~16.9)	0.2	0.80
TpoAb (U/mL)	9.1 (7.0~12.4)	10.1 (8.1~13.0)	10.3 (7.7~12.7)	1.5	0.23
Tg (μg/L)	10.3 (5.8~17.1)	9.2 (6.1~14.6)	9.0 (4.6~14.6)	0.3	0.73
SIC (μg/L)	82.9 (74.2~92.6)	79.0 (69.6~88.4)	76.9 (65.4~88.5)	2.9	0.05
24hour-UIE (μg)	207.1 (155.7~322.0)	262.6 (198.9~350.9)	263.9 (191.0~332.1)	2.2	0.11
Morning UIC (μg/L)	156.8 (106.5~236.2)	177.0 (120.8~239.7)	145.3 (116.7~216.4)	0.9	0.42

-, no comparison.

**Table 2:** Characteristics of the healthy pregnant women and the pregnant women with different thyroid disorders.

Indicators	Healthy	TD1	TD2	TD3	TD4	TD5	*S*	*P*
*n*	429	12	24	15	23	56	–	–
Age (year)	30.7 ± 3.7	30.6 ± 2.9	31.4 ± 3.9	31.6 ± 3.3	29.4 ± 3.2	31.1 ± 3.4	1.0	0.42
Weight (kg)	60.3 ± 11.4	61.0 ± 13.1	58.8 ± 10.1	63.1 ± 8.7	54.5 ± 7.1	61.8 ± 10.4	1.8	0.12
BMI (kg/m^2^)	22.4 ± 4.6	22.4 ± 4.2	21.8 ± 3.5	23.7 ± 2.8	20.7 ± 2.8	23.1 ± 3.6	1.3	0.26
PW (week)	20.5 (13.0~27.0)	17.1 (10.5~19.3)	13.2 (12.2~16.6)	14.6 (12.6~28.1)	27.2 (13.2~28.1)	24.0 (13.0~26.3)	15.0	0.01
TSH (mU/L)	1.9 ± 0.9	6.7 ± 4.9*	0.1 ± 0.1*	2.3 ± 1.2	1.5 ± 0.8	2.0 ± 0.9	52.7	<0.01
FT3 (pmol/L)	4.6 ± 0.6	4.7 ± 0.6	6.4 ± 2.8*	4.4 ± 0.6	4.8 ± 0.8	4.7 ± 0.6	21.4	<0.01
FT4 (pmol/L)	14.3 ± 2.4	14.5 ± 1.8	21.5 ± 9.1*	10.6 ± 2.3*	17.8 ± 3.0*	14.5 ± 2.7	35.4	<0.01
TgAb (U/mL)	15.1 (13.4~17.3)	17.0 (13.5~21.0)	16.6 (13.1~27.7)	13.6 (11.5~17.4)	16.6 (14.3~32.1)*	116.1 (47.7~249.9)*	124.5	<0.01
TpoAb (U/mL)	9.9 (7.7~12.7)	10.6 (8.7~26.3)	10.0 (7.114.7)	8.8 (6.9~10.5)	11.6 (10.4~14.2)*	81.0 (35.3~166.7)*	103.9	<0.01
Tg (μg/L)	9.4 (5.6~15.4)	12.4 (5.6~18.1)	8.8 (4.7~24.9)	6.8 (6.3~8.3)	6.6 (2.7~13.1)	1.6(0.2~8.6)*	44.9	<0.01
SIC (μg/L)	79.6 (70.1~90.54)	78.4 (74.3~94.0)	103.3 (88.6~111.4)*	59.2 (52.5~79.9)*	86.3 (82.7~112.2)*	88.3 (74.6~100.7)*	44.1	<0.01
24hour-UIE (μg)	251.3 (176.0~340.4)	226.8 (179.1~250.2)	141.9 (91.2~213.7)*	250.8 (179.1~283.2)	222.5 (164.6~283.6)	210.7 (139.9~218.7)	11.3	0.02
Morning UIC (μg/L)	158.7 (114.5~230.9)	212.5 (166.1~320.7)	98.6 (80.1~147.0)*	147.0 (107.5~193.4)	152.3 (125.4~168.2)	160.1 (118.6~216.6)	11.9	0.02

PW, pregnant week; SIC, serum iodine concentration; 24hour-UIE, 24 hour urine iodine excretion; TD1, hypothyroidism or subclinical hypothyroidism; TD2, hyperthyroidism or subclinical hyperthyroidism; TD3, hypothyroxinemia; TD4, hyperthyroxinemia; TD5, autoimmune thyroiditis. *indicated P ≤ 0.05 compared with healthy pregnant women. -, no comparison.

The median SIC for euthyroid pregnant women was 79.6 μg/L; specifically, it was found to be 82.9 μg/L in the first trimester, 79.0 μg/L in the second trimester, and 76.9 μg/L in the third trimester. Notably, a gradual decline in the median SIC was observed as pregnancy progressed (*P ^for trend^* < 0.05). Significant variations were observed in SIC among healthy pregnant women and those with different thyroid disorders.

The median 24-hour UIE among pregnant women with normal thyroid function was recorded as 251.3 μg. Specifically, it was observed to be 207.1 μg during the first trimester, 262.6 μg during the second trimester, and 263.9 μg during the third trimester. It is worth noting that the change trend demonstrated an inverse relationship with that of SIC, exhibiting a gradual increase in 24-hour UIE as pregnancy progressed (*P ^for trend^* < 0.05). Furthermore, euthyroid pregnant women exhibited significantly higher levels of 24-hour UIE compared to those with hyperthyroidism or subclinical hyperthyroidism.

The Tg levels of euthyroid pregnant women were measured to be 9.4 μg/L, with no significant fluctuations observed throughout the different trimesters. Additionally, it was found that Tg levels varied between euthyroid pregnant women and those with autoimmune thyroiditis.

The mean neonatal TSH level of the offspring was 3.3 mU/L. No significant differences were observed in neonatal outcomes and neonatal TSH values between pregnant women with and without thyroid disorders. When analyzed separately based on different types of thyroid disorders, the results also indicated no statistically significant difference in the aforementioned indicators (**Table 3**).

**Table 3:** Characteristics of the offspring.

Indicators	All	euthyroid pregnant women	Pregnant women with thyroid disorders						S	P
			All	TD1	TD2	TD3	TD4	TD5		
*n*	559	429	130	12	24	15	23	56	–	–
Delivery week (week)	39.4 (38.4~40.1)	39.4 (38.5~40.1)	39.3 (38.3~40.3)	39.1(37.4~40.2)	39.0 (38.4~39.8)	39.3 (36.1~40.4)	39.4 (37.4~40.1)	39.5 (39.0~40.4)	5.0	0.4131
Delivery mode (normal/cesarean)	238/213	188/237	50/76	6/6	7/17	3/10	11/12	23/30	NA	0.4701
Gender (male/female)	286/229	225/196	61/63	9/2	9/15	4/9	12/10	27/26	NA	0.1278
Length (cm)	50 (50~51)	50 (50~51)	50 (50~51)	50 (49~50)	50 (50~51)	50 (48~51)	50 (49~50)	50 (50~51)	4.8	0.4447
Weight (kg)	3.4 ± 0.5	3.5 ± 0.5	3.4 ± 0.6	3.1 ± 0.8	3.5 ± 0.4	3.3 ± 0.8	3.2 ± 0.7	3.4 ± 0.4	1.8	0.1030
Head circumference (cm)	34.4 ± 1.2	34.4 ± 1.2	34.3 ± 1.4	34.3 ± 1.0	34.5 ± 0.8	34.6 ± 1.8	33.9 ± 1.7	34.3 ± 1.4	0.8	0.5869
Neonatal TSH (mU/L)	3.3 ± 2.3	3.3 ± 2.4	3.2 ± 1.8	3.3 ± 1.8	3.1 ± 1.7	3.3 ± 1.5	3.6 ± 2.0	3.1 ± 1.9	0.3	0.9643
Apgar score (≥8/<8)	550/9	423/6	127/3	11/1	23/1	10/0	22/1	56/0	NA	0.1445

TD1, hypothyroidism or subclinical hypothyroidism; TD2, hyperthyroidism or subclinical hyperthyroidism; TD3, hypothyroxinemia; TD4, hyperthyroxinemia; TD5, autoimmune thyroiditis. -, no comparison. NA, by Fisher's exact test.

### 3.2 Correlation analysis on maternal iodine nutrition, thyroid function and neonatal TSH

**Figure 1:**
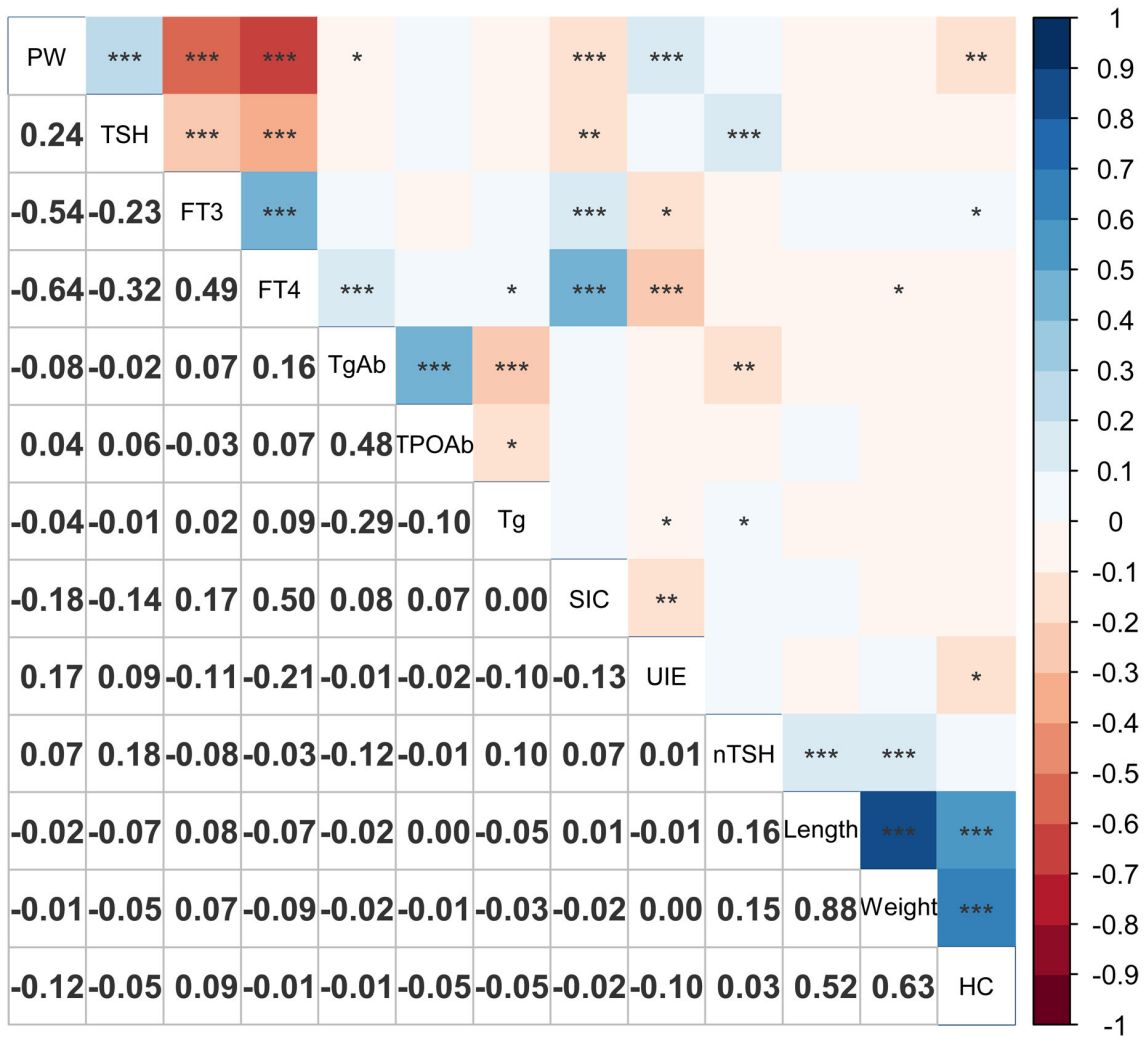
Correlation plot of maternal thyroid function, iodine nutrition and offspring’s indicators. *0.01 < *P* ≤ 0.05; **0.001 < *P* ≤ 0.01; ***P ≤ 0.001. PW, pregnant week; SIC, serum iodine concentration; UIE, 24-hour urine iodine excretion; nTSH, neonatal TSH; HC, head circumference.

The association between iodine nutritional status in pregnant women and their thyroid function, as well as its correlation with neonatal TSH levels, was investigated. The results are presented in **Figure 1**.

The maternal SIC exhibited significant positive correlations with FT3, FT4, and TgAb levels, and a significant negative correlation with gestational age, TSH levels, and 24-hour UIE.

It was worth noting that neonatal TSH levels exhibited a significant positive correlation with maternal TSH and Tg, while displaying a significant negative correlation with TgAb, although the correlation coefficients was not substantial.

### 3.3 Association between iodine nutritional status, thyroid function indicators and thyroid disorders in pregnant women

#### 3.3.1 Effect of maternal iodine nutrition on thyroid function of pregnant women

Given the pivotal role of FT4 as a crucial thyroid hormone in human physiology, a liner regression was conducted in this study to further examine the impact of iodine nutritional status on FT4 levels across different trimesters of pregnancy. After adjusting for mother’s age, pregnancy week, thyroid disorders, and the other indicators list in **Table 4**, the results revealed a significant impact of SIC on maternal FT4 levels across all three trimesters, with varying degrees of importance observed in each trimester. The results also showed that the TSH level emerged as the primary determinant of FT4 during the first trimester, while SIC exerted a predominant influence on FT4 levels in the second and third trimesters, after adjusting for the confounders listed in **Table 4**. Significant effect of thyroid disorders was observed during the third trimester.

**Table 4:** Analysis of the effect of iodine nutritional status on FT4 in pregnant women.

Indicators	T1			T2			T3		
	*β’*	*t*	*P*	*β’*	*t*	*P*	*β’*	*t*	*P*
Age	-0.12	-1.61	0.1099	-0.19	-3.19	0.0017	-0.24	-3.35	0.0011
Pregnant week	0.03	0.34	0.7372	-0.31	-5.10	<0.0001	-0.11	-1.46	0.1473
TSH	-0.37	-4.95	<0.0001	-0.05	-0.77	0.4413	-0.10	-1.42	0.1593
SIC	0.23	3.06	0.0027	0.42	6.94	<0.0001	0.51	7.09	<0.0001
24-hour UIE	-0.01	-0.19	0.8484	-0.12	-2.02	0.0448	0.06	0.82	0.4155
Thyroid disorders	0.11	1.52	0.1307	0.08	1.41	0.1597	0.25	3.44	0.0008

T1, the first trimester; T2, the second trimester; T3, the third trimester; SIC, serum iodine concentration; 24hour-UIE, 24 hour urine iodine excretion.

#### 3.3.2 The relationship between iodine nutritional levels and thyroid disorders in pregnant women

**Figure 2:**
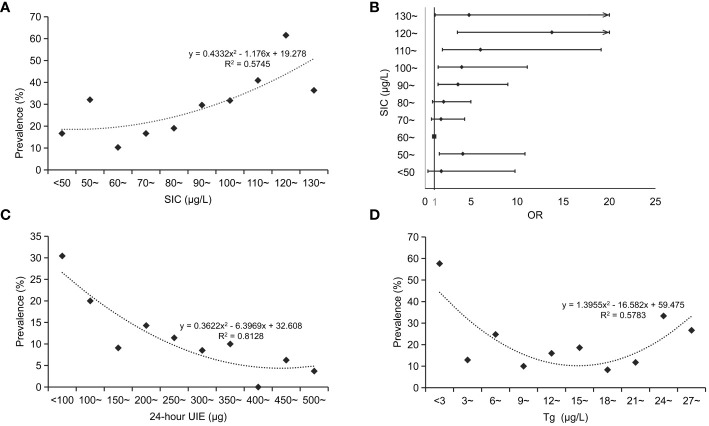
Relationship between serum iodine levels and thyroid disorders in pregnant women. **(A)**, relationship between SIC and prevalence of thyroid disorders in pregnant women; **(B)**, changes in the risk of thyroid disorders at varying SIC among pregnant women; **(C)**, relationship between 24-hour UIE and prevalence of thyroid disorders in pregnant women; **(D)**, relationship between Tg and prevalence of thyroid disorders in pregnant women. SIC, serum iodine concentration; OR, odd ratio; 24-hour UIE, 24-hour urine iodine excretion.

We further investigated the association between SIC and thyroid disorders in pregnant women. The results revealed an initially decreasing and subsequently increasing with rising SIC levels. Notably, when SIC nearly 60 ~ 70 μg/L, a relatively lower prevalence of thyroid disorders was observed (**Figure 2A**). The logistic regression analysis also revealed a significant positive correlation between the prevalence of thyroid disorders and SIC, with higher OR values observed in both sides of SIC compared to the reference group (SIC in the range of 60 ~ 70 μg/L), after adjusted for mother’s age and trimesters, **Figure 2B**.

For the 24-hour UIE, the prevalence of thyroid disorders in pregnant women was lower when the value was between 250 μg and 450 μg (**Figure 2C**). For the Tg, the prevalence of thyroid disorders in pregnant women was comparatively lower when Tg levels ranged between 9 and 21 μg/L (**Figure 2D**).

### 3.4 Maternal factors affected on neonatal TSH

Both high and low TSH levels in neonates are considered abnormal. Quantile regression analysis was employed to examine the impact of maternal iodine nutrition and thyroid function on the different quantiles of neonatal TSH concentrations in pregnant women with and without thyroid disorders respectively. The findings revealed that in euthyroid pregnant women, maternal TSH exhibited a notable influence on neonatal TSH levels, particularly at the *25th*, *50th* and *75th* quantiles, after adjusting for mother’s age, pregnant weeks, Tg levels and TPOAb levels (**Table 5**). For the *25th*, *50th*, and *75th* quantiles, the increase in maternal TSH was accompanied by an increase in neonatal TSH. In the *50th* quantile, the increase of maternal TSH was accompanied by the most significant increase of neonatal TSH. However, maternal TSH had no significant effect on neonatal TSH in pregnant women with thyroid disorders (**Table 5**).

**Table 5:** Effect of maternal TSH (adjusted for iodine status) on neonatal TSH.

neonatal TSH quantile	Euthyroid pregnant women		Pregnant women with thyroid disorders	
	*β*	*t*	*β*	*t*
*P10*	<0.001	<0.001	0.027	0.429
*P25*	0.237	2.615**	-0.006	-0.088
*P50*	0.434	3.945***	-0.005	-0.057
*P75*	0.412	2.752**	0.201	1.419
*P90*	0.342	1.483	-0.058	-0.289

**0.001 < P ≤ 0.01; ***P ≤ 0.001.

### 3.5 The appropriate metric for assessing iodine nutrition

**Figure 3:**
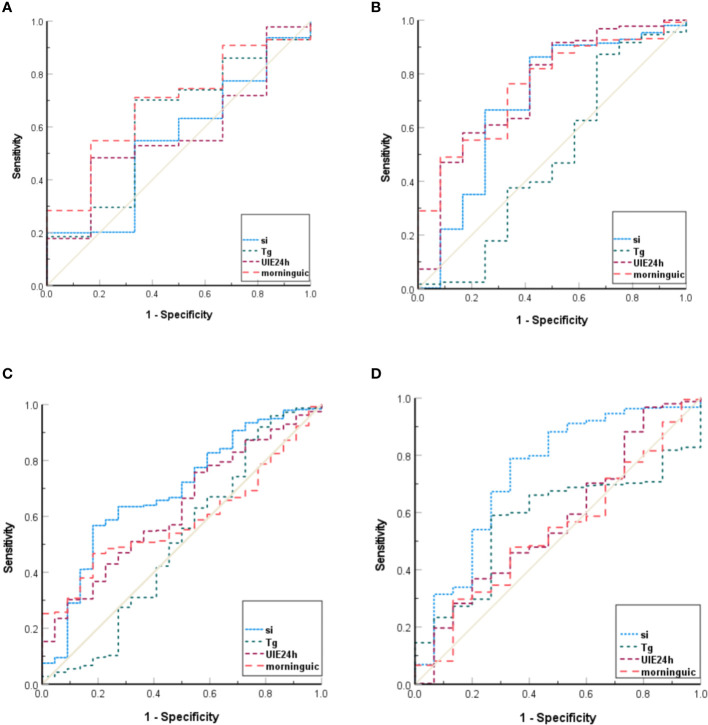
ROC curves for assessing TSH, FT4. **(A)**, ROC curves for high TSH (AUC _SIC_=0.549, AUC _24-hour UIE_=0.573, AUC _morning UIC_=0.688, AUC _Tg_=0.619); **(B)**, ROC curves for low TSH (AUC _SIC_=0.697, AUC _24-hour UIE_=0.747, AUC _morning UIC_=0.753, AUC _Tg_=0.516); **(C)**, ROC curves for high FT4 (AUC _SIC_=0.687, AUC _24-hour UIE_=0.627, AUC _morning UIC_=0.585, AUC _Tg_=0.508); **(D)** ROC curves for low FT4 (AUC _SIC_=0.737, AUC _24-hour UIE_=0.569, AUC _morning UIC_=0.533, AUC _Tg_=0.574).

Iodine deficiency or excess initially leads to abnormal levels of TSH and FT4. ROC curves were constructed based on TSH and FT4 (below or above the reference limits), and the AUC values were calculated. The findings demonstrated that morning UIC was a better indicator for high/low TSH, whereas SIC was a better indicator for high/low FT4 among pregnant women (**Figure 3**).

## 4 Discussion

This study was conducted in Harbin, a northern city in China, which served as a representative iodine-adequate area for the general population in China. Nowadays, with the better and better implementation of USI around the world, such areas are gradually increasing (18). In this study, our aim was to assess the iodine nutritional status and thyroid function of pregnant women across different trimesters in this type of area, analyze the variations in iodine nutritional status and thyroid function levels during pregnancy, and investigate their association with thyroid disorders. Additionally, we evaluated the neonatal outcomes of the subjects to examine the impact of iodine nutritional status and thyroid function on these outcomes and their related influencing factors. To our best knowledge, this study provides a comprehensive evaluation of iodine nutritional status among pregnant women throughout pregnancy.

Although UIC is the most commonly used biomarker for assessing iodine status in populations, UIC only reflects recent iodine intake (19). It can be affected by water intake and diet, especially during pregnancy, as excessive water intake leads to an increased urine output and dilution of iodine concentrations (20). In addition to UIC, thyroid volume, thyroid function, and SIC have also been used as sensitive biomarkers to assess the iodine nutrition status (12, 14, 19, 21). However, the thyroid volume can only reflect longer-term iodine nutrition status; thyroid function values can be maintained within the normal reference range in the presence of inadequate iodine intake owing to tight homeostatic regulation (15). By contrast, the SIC is a key biomarker of iodine metabolism, as serum iodine is controlled by intrinsic mechanisms. However, long-term inadequate iodine status will break the iodine balance, leading to abnormal serum iodine levels and ultimately thyroid dysfunctions and diseases. Thus, the SIC may be a potential candidate as a biomarker for the evaluation of individual iodine status. In this study, there were some discrepancies among SIC, 24-hour UIE and morning UIC. These discrepancies may be attributed to their own characteristics. SIC served as a relatively stable indicator that reflects an individual’s iodine nutrition status over a relatively long period of time (22). The 24-hour UIE can also reflect the iodine nutritional level of the human body from the perspective of intake (23). Morning UIC served as an indicator of recent iodine intake, reflecting the individual’s current iodine nutritional status (24). Additionally, in circumstances where body iodine balance is maintained without any imbalances, ingested iodine has the potential to be stored within the blood and thyroid gland reservoirs, leading to a decrease in excretion (25).

In this study, the sample size for each trimester pregnancy was limited. However, in order to provide the clue for future studies, a preliminary analysis was conducted on the variations of iodine nutrition indicators during different stages of pregnancy. Our pilot results showed SIC levels exhibited a gradual decrease trend, while 24-hour UIE remained quite stable. During early pregnancy, there is a significant rise in hCG, which also leads to a mild increase in FT3 and FT4 (26, 27). Consequently, SIC, the essential substrate for thyroid hormone synthesis, will be elevated as well. This increased demand subsequently results in reduced 24-hour UIE. However, no significant alteration was observed in the morning UIC. The morning UIC exhibited considerable variability and displayed a significantly skewed distribution, implying that the detecting of differences and trends may require a relatively large sample size. In addition, from the perspective of iodine nutrition evaluation, this study revealed that the prevalence of thyroid disorders in pregnant women was significantly lower when SIC in the range of 60 ~ 70 μg/L, 24-hour UIE in the range of 250 ~ 450 μg, and Tg in the range of 9 ~ 21 μg/L. Notably, these ranges were observed in regions with adequate iodine nutrition for the general population as well as areas where iodine supplementation was implemented. The suitable range of SIC in this study was basically consistent with the research findings of Ji et al. (6) The suitable 24-hour UIE value exceeded that reported in Saudi Arabia (28). The suitable of Tg were lower than those documented in many studies (29). The discrepancy may be mainly attributed to the fact that our research area was characterized by appropriate iodine nutrient levels, as well as differences in study design. Consequently, our findings will offer valuable insights for evaluating iodine nutritional status among pregnant women residing in countries where USI programs are increasingly being promoted.

The results of this study revealed significant differences in Tg, SIC, 24-hour UIE, and morning UIC among healthy pregnant women and pregnant women with different types of thyroid disorders (primarily excluding thyroid nodules). The results of other studies have shown a similar trend. For example, one study conducted in Shanghai (China) displayed that both low and high urine iodine concentration was related to thyroid function and autoimmunity, it is important to keep an optimal iodine nutrition status during gestation (5). It is worth noting that the impact of iodine nutritional status on the occurrence of thyroid diseases is widely recognized, as inadequate iodine intake hampers the maintenance of normal physiological morphology and function of the thyroid gland. Nevertheless, it should be noted that thyroid abnormalities can also disrupt iodine metabolism within the body (30–32). Therefore, further mechanistic investigations are warranted to ascertain the causality between thyroid disorders and abnormal changes in iodine nutrition indicators. Additionally, the study findings indicated no significant difference in neonatal outcomes between offspring of euthyroid pregnant women and those with thyroid disorders. The results suggest that thyroid disorders in this study does not affect neonatal outcomes.

The physiological roles of thyroid hormones encompass various functions, playing a crucial role in the maintenance of regular pregnancy and optimal development of the fetus. Maternal levels of TSH and FT4 exhibit dynamic fluctuations throughout gestation. Our preliminary results revealed that after adjusting for mother’s age, pregnancy weeks and thyroid disorders, maternal TSH levels exhibited an elevation trend from the first trimester to the third trimester. Conversely, there was a contrasting pattern observed in maternal FT3 and FT4 levels. The findings of this study also demonstrated that both TSH and SIC exert an influence on FT4 levels in the first trimester after adjusting for the confounders. In the second trimester, FT4 levels are influenced by SIC and 24-hour UIE. During the third trimester, SIC and thyroid disorders predominantly affects FT4 levels. These results indicate that maintaining a normal iodine nutritional status, particularly with regards to serum iodine levels, is crucial for the optimal development of pregnant women and their offspring throughout pregnancy. However, due to the limited sample size per pregnancy, it is imperative to further validate the findings of this comparison in a larger population. Furthermore, changes in standardized partial regression coefficients across different trimesters suggest that SIC level in the third trimester plays a more significant role in maintaining normal thyroid function compared to other periods. Conversely, TSH appears to have a greater impact during the first trimester. This observation aligns with alterations observed in thyroid function during pregnancy (3, 33).

TSH is a crucial biomarker in detecting thyroid dysfunction and has a significant correlation with neurodevelopmental outcomes. The results of the present study showed that neonatal TSH values were significantly correlated with maternal TSH, and the quantile regression results showed that TSH had an nonlinear effect on neonatal TSH, especially on the 25th, 50th and 75th, which was in accordance with theoretical knowledge. Lee et al.’s study shown maternal serum TSH in pregnancy was associated with the increased risks of prematurity and neonatal respiratory distress syndrome in offspring (34). Hou et al.’s study also provided epidemiological evidence that maternal serum TSH and FT4, especially during the first trimester, were associated with linear or nonlinear variations in neonatal metabolites (35). In addition, the correlation results showed that neonatal TSH were significantly correlated with Tg, and TgAb, while their effects were not significant enough in quantile regression. This result was consistent with other studies (36, 37). Yuan et al.’s study showed TPOAb positivity was not found to be associated with adverse pregnancy-related or fetal outcomes in euthyroid women. Among euthyroid women carrying a female fetus, TPOAb positivity exhibited a strong correlation with preterm births (36). However, for female infants, our quantile regression results also showed that the effect of positive TPOAb on neonatal TSH was not significant (data not shown). For Tg, it serves as an indicator of iodine nutrition and also could reflect the levels of thyroid hormones. Our findings demonstrated a significant association between Tg levels and neonatal TSH, highlighting the impact of maternal iodine nutrition and thyroid function on neonatal TSH although maternal thyroid disorders had a non-significant effect. Future research could benefit from expanding the sample size to conduct multi-center studies.

Currently, there is ongoing debate regarding the optimal index for assessing individual iodine nutrition in pregnant women. The findings of this study demonstrated that different indicators reflected different types of disorders. For FT4 abnormalities, correlation analysis revealed a stronger association between SIC and FT4 as well as TSH levels in pregnant women compared to the other three indicators. Additionally, when identifying abnormal levels of FT4, SIC demonstrated a higher AUC values than morning UIC, 24-hour UIE and Tg. For TSH abnormalities, morning UIC showed a better discrimination effect. Considering that abnormal iodine nutrition primarily affects FT4, followed by TSH, SIC may serve as a better indicator for evaluating iodine nutrition in pregnant women.

The objective of this study was to investigate the impact of iodine nutrition on maternal and fetal health during pregnancy. Despite evaluating neonatal outcomes, certain limitations still existed. Firstly, although pregnant women were monitored for their offspring’s outcomes, there was no continuous follow-up of the same woman throughout different trimesters. Second, the number of pregnant women in each trimester was limited, and the results still need to be analyzed and verified in a larger sample. Furthermore, while SIC and 24-hour UIE were utilized as indicators of the individual iodine nutritional status among pregnant women, a comprehensive investigation regarding dietary iodine intake was not conducted.

In conclusion, the iodine nutrition level differs between healthy pregnant women and those with thyroid disorders. Iodine nutrition status of pregnant women exerted an impact on their thyroid functions, with varying magnitudes observed across different stages of pregnancy. The neonatal TSH were correlated with and affected by maternal TSH in euthyroid pregnant women.

## Data availability statement

The original contributions presented in the study are included in the article/Supplementary material, Further inquiries can be directed to the corresponding author/s.

## Ethics statement

The studies involving humans were approved by the ethics committees of Harbin Medical University (HRBMUCEDC20211002). This study was conducted according to the provisions of the Declaration of Helsinki. Written informed consent was obtained from all participants or their guardian. The studies were conducted in accordance with the local legislation and institutional requirements. Written informed consent was obtained from the individual(s), and minor(s)’ legal guardian/next of kin, for the publication of any potentially identifiable images or data included in this article.

## Author contributions

LF: Funding acquisition, Methodology, Writing – original draft. YB: Data curation, Methodology, Writing – review & editing. SC: Methodology, Writing – review & editing. SW: Investigation, Writing – review & editing. WZ: Data curation, Writing – review & editing. YH: Investigation, Writing – review & editing. DS: Conceptualization, Funding acquisition, Writing – review & editing.
